# The Role of Age in Predicting the Outcome of Caustic Ingestion in Adults: A Retrospective Analysis

**DOI:** 10.1186/1471-230X-11-72

**Published:** 2011-06-14

**Authors:** Jui-Min Chang, Nai-Jen Liu, Betty Chien-Jung Pai, Yun-Hen Liu, Ming-Hung Tsai, Ching-Song Lee, Yin-Yi Chu, Chih-Chuan Lin, Cheng-Tang Chiu, Hao-Tsai Cheng

**Affiliations:** 1 Division of Digestive Therapeutic Endoscopy, Department of Gastroenterology and Hepatology, Chang Gung Memorial Hospital, Linkou Branch, Tao-Yuan, Taiwan; 2 Chang Gung University College of Medicine, Tao-Yuan, Taiwan; 3 Department of Gastroenterology and Hepatology, Chang Gung Memorial Hospital, Linkou Branch, Tao-Yuan, Taiwan; 4 Thoracic & Cardiovascular Surgery, Chang Gung Memorial Hospital, Linkou Branch, Tao-Yuan, Taiwan; 5 Department of Orthodontics and Craniofacial Dentistry, Chang Gung Memorial Hospital, Taipei, Taiwan; 6 Department of Emergency Medicine, Chang-Gung Memorial Hospital, Taoyuan, Taiwan; 7 Department of Gastroenterology, Shin Kong Wu Ho-Su Memorial Hospital, Taipei, Taiwan

## Abstract

**Background:**

Although the outcomes of caustic ingestion differ between children and adults, it is unclear whether such outcomes differ among adults as a function of their age. This retrospective study was performed to ascertain whether the clinical outcomes of caustic ingestion differ significantly between elderly and non-elderly adults.

**Methods:**

Medical records of patients hospitalized for caustic ingestion between June 1999 and July 2009 were reviewed retrospectively. Three hundred eighty nine patients between the ages of 17 and 107 years were divided into two groups: non-elderly (< 65 years) and elderly (≥ 65 years). Mucosal damage was graded using esophagogastroduodenoscopy (EGD). Parameters examined in this study included gender, intent of ingestion, substance ingested, systemic and gastrointestinal complications, psychological and systemic comorbidities, severity of mucosal injury, and time to expiration.

**Results:**

The incidence of psychological comorbidities was higher for the non-elderly group. By contrast, the incidence of systemic comorbidities, the grade of severity of mucosal damage, and the incidence of systemic complications were higher for the elderly group. The percentages of ICU admissions and deaths in the ICU were higher and the cumulative survival rate was lower for the elderly group. Elderly subjects, those with systemic complications had the greatest mortality risk due to caustic ingestion.

**Conclusions:**

Caustic ingestion by subjects ≥65 years of age is associated with poorer clinical outcomes as compared to subjects < 65 years of age; elderly subjects with systemic complications have the poorest clinical outcomes. The severity of gastrointestinal tract injury appears to have no impact on the survival of elderly subjects.

## Background

Caustic agents are known to provoke a wide range of gastrointestinal (GI) tract injuries with long-term complications; such complications include esophageal stricture and the development of esophageal carcinoma [1]. Esophagogastroduodenoscopy (EGD) is the gold standard for safe assessment of the depth and extent of caustic ingestion injury and for determination of the appropriate regimens to treat such injuries. The appropriate timing of EGD and the risks associated with performance of this procedure are well established [2-8].

Caustic agents with pH values < 2 or > 12 are extremely corrosive to the GI tract. The extent of tissue destruction by such agents is dependent on multiple variables such as type, physical form, and concentration [5]. In the United States, the estimated incidence of caustic injuries ranges between 5,000 to 15,000 cases per year [9], with alkaline materials being the most commonly encountered. However, in developing countries such as India, hydrochloric and sulfuric acids are the caustic materials most commonly encountered [4,9].

Caustic ingestion in children is primarily accidental and is observed most commonly in children less than 6 years of age [2]. In adults, by contrast, caustic ingestion primarily serves as a suicide modality; in such cases the injuries tend to be more severe [3]. The widespread availability in the United States of highly caustic household products, such as window and drain cleaners and dishwashing agents, has increased the number of accidental caustic injuries in the pediatric population as well as the number of suicide attempts in the adult population [10]. Consequently, studies focusing on the role of age in survival following caustic ingestion are warranted and are expected to yield findings of clinical significance.

The clinical outcomes of caustic ingestion are assumed to be more serious for elderly as opposed to non-elderly subjects but such outcomes are only rarely investigated. The present study was undertaken to compare the clinical outcomes of caustic ingestion injury in elderly and non-elderly adults with regard to gender, intent of ingestion, substance ingested, systemic and GI complications, severity of mucosal injury, and time to expiration.

## Patients and Methods

### Patients

Informed written consent was obtained from all subjects in this study, which was performed according to the guidelines of our institutional review board (IRB). Approval for this retrospective study (CGMH98-3352B) was obtained from our IRB.

The medical records from 389 patients (ranging in age from 17 to 107 years) who were admitted to the Department of Gastroenterology, Chang Gung Memorial Hospital, Tao-Yuan, Taiwan, for caustic ingestion between June 1999 and July 2009 were reviewed. Patients were admitted to the Department of Gastroenterology from the Emergency Room (ER). EGD with a standard upper GI endoscope was performed on all patients within 24 h of ingestion by physicians experienced with this technique. The endoscopes used in this study were the Olympus GIF XQ-230, the GIF Q 240X, and the GIF Q-260; the diameters of these endoscopes are 9.2, 9.4, and 9.2 mm, respectively (Olympus, Tokyo, Japan). Extreme care was used when passing the endoscope through the injured segment. If perforation was suspected, the procedure was stopped immediately and the endoscope withdrawn.

### Parameters

Subjects were divided into two groups according to age: the non-elderly (< 65 years) and elderly (≥65 years) groups. The parameters investigated in these two groups included gender, intent of ingestion, substance ingested, systemic and GI complications, psychological and systemic comorbidities, severity of mucosal injury as assessed by EGD, and time to expiration. Systemic complications included liver damage, renal insufficiency, disseminated intravascular coagulation (DIC), and hemolysis. Liver damage was defined as a three-fold elevation in the concentration of serum alanine aminotransferase or aspartate aminotransferase above the upper limit of normal. Renal insufficiency was defined as a plasma creatinine concentration greater than 1.4 mg/dL in the absence of other renal diseases. Criteria for DIC were prolonged plasma coagulation time, decreased fibrinogen or antithrombin values, and decreased platelet count. Upper GI complications included bleeding, perforation, and stricture formation. Bleeding was defined as melena, hematemesis, and/or coffee ground vomitus. Perforation was diagnosed by the presence of free air on a plain chest radiograph. Stricture was defined as dysphagia, symptoms of regurgitation, or difficulty in swallowing with confirmation by endoscopy, esophagogram, and/or upper GI radiography.

Mucosal damage was graded according to the modified endoscopic classification of Zargar *et al*. [8] as follows: grade 0 = normal examination; grade 1 = edema and hyperemia of the mucosa; grade 2a = superficial ulceration, erosions, friability, blisters, exudates, hemorrhages, or whitish membranes; grade 2b = grade 2a plus deep, discrete or circumferential ulcerations; grade 3a = small scattered areas of multiple ulcerations and areas of necrosis with brown-black or grayish discoloration; grade 3b = extensive necrosis.

### Immediate management

Blood tests, including complete blood and differential counts, and measurements of alanine transaminase, creatinine, Na^+^, and K^+ ^were performed in the ER. Based on the judgment of the ER physician in charge, patients were treated with a proton pump inhibitor, H_2 _antagonist, or sucralfate gel. Oral intake was withheld and parenteral nutrition was provided until evidence was obtained that the condition of the patient had stabilized. Antibiotics were not administered routinely due to national healthcare insurance restrictions. If infection was suspected, empirical antimicrobial therapies (such as a first generation cephalosporin and/or gentamicin or ciprofloxacin) were administered until blood cultures were obtained. If a pulmonary or respiratory complication was suspected, a chest X-ray was performed. Aspiration pneumonia was defined as a lung infection developing after accidental inhalation of foods, liquids, or stomach contents and was diagnosed by chest X-ray; patient history served to confirm that no other form of lung disease was present.

### Further management

If the patient's condition became unstable or respiratory difficulty was encountered, he/she was transferred to the intensive care unit (ICU) for further evaluation. After discharge, each surviving patient was followed at the outpatient clinic for at least 1 month. If the patient had symptoms of dysphagia during follow-up, an upper GI series or EGD was performed to confirm the presence of stricture. Patients with GI or systemic complications were followed until these patients expired. Those with persistent dilatation were followed for over 7 years. The overall range of follow-up times, therefore, was 3 days to more than 7 years.

### Statistical analyses

Demographic and clinical findings are presented as *n *with a percentage (%), with the exception of age which is expressed as the median (Q1-Q3) and as the mean (minimum-maximum). Pearson chi-square or Fisher's exact tests were performed to identify dispersions in the demographics and clinical findings among the two age groups. For time-related data, Kaplan-Meier analysis with a log-rank test was also performed to indicate the cumulative survival rate by age. Furthermore, univariate and multivariate Cox proportional hazard ratio regression models were used to obtain the hazard rate (HR) with a 95% confidence interval and to determine the cumulative survival rate as a function of age and other confounders. Based on the proportion of systemic complications and the Type I error probability associated with these models, the null hypothesis is 0.05 and the observed power is equal to 98.6%. All statistical assessments were considered significant at a *P *value of less than 0.05. Statistical analyses were performed using SPSS 15.0 statistics software (SPSS Inc, Chicago, IL, USA).

## RESULTS

### Age groups and demographic findings

Three hundred eighty nine subjects were enrolled. The non-elderly group was comprised of 321 (82.5%) subjects less than 65 years of age, and the elderly group was comprised of 68 (17.5%) subjects 65 years of age or older. Median and mean ages for all subjects and for the non-elderly and elderly groups are presented in Table 1. The median age with inter-quartiles (Q1-Q3) for all subjects was 40 (29-55) years; median ages with inter-quartiles (Q1-Q3) for the non-elderly and elderly groups were 36 (26-45) and 74 (68-77) years, respectively. The mean age (minimum to maximum ages) for all subjects was 43.25 (17-107) years; mean ages (minimum to maximum ages) for the non-elderly and elderly groups were 36.72 (17-64) and 74.07 (65-107) years, respectively.

**Table 1:** Patient demographics and caustic damage parameters for all subjects and for those in the non-elderly and elderly groups

Variables	Total (*n *= 389)	Non-elderly (< 65 years) (*n *= 321)	Elderly (≧65 years) (*n *= 68)	*P *value
**Median age **(Q1-Q3)	40 (29 - 55)	36 (26 - 45)	74 (68 - 77)	< 0.001*
**Mean age **(minimum-maximum)	43.25 (17-107)	36.72(17-64)	74.07 (65-107)	
**Gender **(male %)	189 (48.6)	165 (51.4)	24 (35.3)	0.016*
**Substance**				0.472
Clean detergent	193 (49.6)	160 (49.8)	33 (48.5)	0.844
HCl	107 (27.5)	90 (28.0)	17 (25.0)	0.610
Other	89 (22.9)	71 (22.1)	18 (26.5)	0.438
**Acid/alkaline**				0.444
Acid	245 (63.0)	206 (64.2)	39 (57.4)	0.290
Alkaline	124 (31.9)	100 (31.2)	24 (35.3)	0.506
Unknown	20 (5.1)	15 (4.7)	5 (7.4)	0.363
**Grade^a^**				0.002*
1	57 (14.7)	52 (16.2)	5 (7.4)	0.061
2a/2b	153 (39.3)	133 (41.4)	20 (29.4)	0.065
3a	61 (15.7)	53 (16.5)	8 (11.8)	0.328
3b	104 (26.7)	73 (22.7)	31 (45.6)	< .001*
**Furthest location reached by endoscope^a^**				0.091
Esophagus	8 (2.1)	8 (2.5)	0 (0)	-
Stomach	31 (8.0)	22 (6.9)	9 (13.2)	0.075
Duodenum	344 (88.4)	287 (89.4)	57 (83.8)	0.191
**Most severe site of damage as observed by endoscopy^a^**				0.422
Esophagus	82 (21.1)	70 (21.8)	12 (17.6)	0.445
Stomach	267 (68.6)	222 (69.2)	45 (66.2)	0.630
Duodenum	37 (9.5)	28 (8.7)	9 (13.2)	0.249

Data other than those regarding age are expressed as *n *(%).

^a^Data were missing for 14 subjects in the grade category, for 3 subjects in the site of damage category, and for 6 subjects in the depth of scope category.

*P value < 0.05.

Demographic characteristics of the two groups are also presented in Table 1. No significant differences regarding the type of ingested substance, the pH of the ingested substance, or the site most severely damaged as observed by endoscopy were observed between the non-elderly and elderly groups. However, the percentage of male subjects in the non-elderly group was higher than that in the elderly group (*P *= 0.016). Additionally, the severity of mucosal damage was found to be greater for subjects in the elderly group (*P *= 0.002 for overall severity). This difference in severity was primarily attributable to the difference between the two groups in the number of subjects with a mucosal damage grade of 3b (*P *< 0.001).

### Comorbidities, complications, treatment of complications, and clinical outcomes

Table 2 presents the comorbidities, complications, treatments, and clinical outcomes for all subjects and for those in the non-elderly and elderly groups. Psychological comorbidities included major depressive disorder and life-related forms of depression, psychoses, addictions, anxiety, schizophrenia, bipolar disorder, and mental retardation. Systemic comorbidities included: hypertension; diabetes mellitus; joint and spine disorders; renal failure; cerebrovascular, cardiovascular, hepatic, pancreatic, or other organ disease; tumors/cancers; and histories of trauma and surgery. "Depression" was the comorbidity most frequently recorded. All systemic and GI complications observed during follow-up were recorded. Surprisingly, the incidence of psychological comorbidity was lower for the elderly group (30.9%) as compared to the younger age group (48.3%; *P *= 0.009). As expected, the incidence of systemic comorbidity was higher for the elderly (73.5%) as compared to the non-elderly (22.7%; *P *< 0.001) group.

**Table 2:** Comorbidities, complications, treatment of complications, and clinical outcomes for all subjects and for those in the non-elderly and elderly groups

Parameter	Total (*n *= 389)	Non-elderly (< 65 years) (*n *= 321)	Elderly (≧ 65 years) (*n *= 68)	*P *value
**Psychological comorbidities^1^**	176 (45.2)	155 (48.3)	21 (30.9)	0.009*
**Systemic comorbiditities^1^**	123 (31.6)	73 (22.7)	50 (73.5)	< 0.001*
**Suicide/accident^1^**				0.699
Suicide	282 (72.5)	234 (72.9)	48 (70.6)	
Accident	107 (27.5)	87 (27.1)	20 (29.4)	
				
**Systemic complications^1^**	73 (18.8)	47 (14.6)	26 (38.2)	< .001*
Aspiration pneumonia^1^	49 (12.6)	33 (10.3)	16 (23.5)	0.003*
Respiratory failure^1^	37 (9.5)	21 (6.5)	16 (23.5)	< .001*
DIC^1^	14 (3.6)	7 (2.2)	7 (10.3)	0.005*
Hepatic^2^	14 (3.6)	11 (3.4)	3 (4.4)	0.719
				
**GI complications^1^**	90 (23.1)	72 (22.4)	18 (26.5)	0.493
Stricture^1^	81 (20.8)	67 (20.9)	14 (20.6)	0.958
Bleeding^2^	17 (4.4)	14 (4.4)	3 (4.4)	1.000
Perforation^2^	11 (2.8)	8 (2.5)	3 (4.4)	0.298
Fistula^2^	5 (1.3)	5 (1.6)	0 (0)	0.592
				
Fever^1^	82 (21.1)	57 (17.8)	25 (36.8)	0.001*
Antibiotic usage^2^	114 (29.3)	81 (25.2)	33 (48.5)	< .001*
Operation^a,1^	63 (16.2)	52 (16.2)	11 (16.2)	0.996
Dilatation^a,1^	35 (9.0)	26 (8.1)	9 (13.2)	0.179
ICU admissions^1^	48 (12.3)	28 (8.7%)	20 (29.4%)	< .001*
Days in the ICU^3^	10.0 (3.2)	9 (2.8)	10.0 (3.5)	0.249
Number of expirations in the ICU^1^	24 (6.2)	12 (3.7)	12 (17.6)	< .001*
Days to expiration in the ICU^3^	16.0 (1.6)	15.0 (1.7)	16.0 (3.6)	0.273

DIC, disseminated intravascular coagulation

^a^Dilatation and operation: management options in the event of complications

Findings are expressed as n (%), except for ICU admissions and days to expiration which are expressed as medians (standard error).

^1^Pearson chi-square test; ^2^Fisher's exact test; ^3^Log-rank test; *P value < 0.05

The incidences of suicide attempts and accidental ingestions were similar for the two groups, with suicide attempts more commonly involved.

Systemic complications included aspiration pneumonia, respiratory failure, DIC, and hepatic complications. The overall incidence of these complications was higher in the elderly than in the non-elderly group (*P *< 0.001). The differences in incidences of aspiration pneumonia, respiratory failure, and DIC between the two groups were statistically significant (*P *values of 0.003, < 0.001, and 0.005, respectively). By contrast, the incidence of hepatic complications did not differ between the two groups (*P *= 0.719). Fever developed in 21.1% of all subjects, in 17.8% of those in the non-elderly group, and in 36.6% of those in the elderly group; the difference between the two groups was significant (*P *= 0.001).

Upper GI complications included bleeding, perforation, stricture formation, and fistula formation. The overall incidence of GI complications and the incidences of each of these complications did not differ significantly between the two groups.

Treatments were selected according to the complication manifested and the experience of the treating physician(s). These procedures included the most appropriate surgery and/or dilatation and administration of antibiotics. Surgical procedures included feeding jejunostomy, subtotal gastrectomy, esophagectomy, esophageal reconstruction, and the Whipple operation for perforation. If a stricture formed, patients received dilatation or/and surgery. In the event that bleeding occurred, EGD was repeated if possible; some patients died suddenly following hematemesis. If the event of perforation, a surgeon was consulted immediately; some patients expired suddenly after diagnosis by an imaging technique such as chest X-ray. The incidences of surgical treatments and of dilatation did not differ significantly between the two groups. If infection was suspected, empirical antimicrobial therapy was administered immediately. More specific therapy was administered when the infectious organism was identified. Antibiotics were administered to 29.3% of all subjects, to 25.2% of those in the non-elderly group, and to 48.5% of those in the elderly group; the difference between the two groups was significant (*P *< 0.001).

Admission to the ICU was required for 29.4% of patients in the elderly group as compared to 8.1% of patients in the non-elderly group; the difference in admissions was significant (*P *< 0.001). Furthermore, the percentage of patients who expired following admission to the ICU was greater for the elderly group than for the non-elderly group (17.6% versus 3.7%; *P *< 0.001). However, no differences between the two groups regarding days in the ICU or time to expiration in the ICU were observed. Causes of mortality for the non-elderly group were: DIC (3 cases), hemotypsis (4 cases), tracheal perforation (1 case), and respiratory failure (4 cases). Causes of mortality for the elderly group were: DIC (2 cases), hemotypsis (1 case), perforation (1 case), and respiratory failure (8 cases).

### Predictors of survival

Table 3 presents the results of univariate and multivariate Cox proportional hazard ratio regression models of mortality risk considering relative age-related confounding factors. By univariate analysis, the elderly group was found to have a greater mortality risk as compared to the non-elderly group (HR = 5.63; 95% CI for HR = 2.48 - 12.76; *P *< 0.001). Apart from age, other variables correlating with increased mortality risk based on univariate analysis in order of decreasing HR were: systemic complications (HR = 26.44; 95% CI = 7.69 - 90.90; *P *< 0.001), antibiotic usage (HR = 14.12; 95% CI = 4.13 - 48.19; *P *< 0.001), fever (HR = 7.36; 95% CI = 2.96 - 18.25; *P *< 0.001), intended suicide (HR = 4.34; 95% CI = 1.02 - 18.50; *P *= 0.047), GI complications (HR = 2.85; 95% CI = 1.26 - 6.48; *P *= 0.012), hepatic complications (HR = 2.37; 95% CI = 1.29 - 4.36; *P *= 0.006), respiratory failure (HR = 2.14; 95% CI = 1.35 - 3.39; *P *= 0.001), grade 2a/2b injury (HR = 0.19; 95% CI = 0.06 - 0.57; *P *= 0.003) and grade 3a injury (HR = 0.12, 95% CI = 0.02 - 0.87; *P *= 0.037). The HR for grade 1 injury was 0 because no patients in this category expired.

**Table 3:** Univariate and multivariate Cox proportional hazard ratio regression model of mortality risk considering relative age-related confounding factors

	Univariate			Multivariate		
	
Variables	HR	(95% CI for HR)	*P *value	HR	(95% CI for HR)	*P *value
**Age**						
< 65 years	(Reference)			(Reference)		
≧65 years	5.63	(2.48 - 12.76)	< .001*	1.09	(0.80 - 1.48)	0.585
Gender^1^						
Females	(Reference)			(Reference)		
Males	1.94	(0.84 - 4.51)	0.122	1.24	(0.98 - 1.57)	0.070
**Substance^1^**						
Clean detergent	1.79	(0.43 - 7.49)	0.428	-		
HCl	2.72	(0.79 - 9.38)	0.114	-		
Other	(Reference)			-		
Acid/alkaline^1^						
Acid	(Reference)			-		
Alkaline	1.36	(0.57 - 3.24)	0.485	-		
Unknown	1.84	(0.47 - 8.23)	0.426	-		
**Grade^1^**						
1	0	(0 - 7.75 × 10^302^)	0.970	1.90	(1.29 - 2.80)	0.001*
2a/2b	0.19	(0.06 - 0.57)	0.003*	0.88	(0.64 - 1.20)	0.404
3a	0.12	(0.02 - 0.87)	0.037*	1.18	(0.82 - 1.70)	0.366
3b	(Reference)			(Reference)		
**Most severe site of damage observed by endoscopy^1^**						
Esophagus	(Reference)			-		
Stomach	1.00	(0.33 - 3.08)	0.994	-		
Duodenum	2.92	(0.78 - 10.86)	0.111	-		
**Furthest location reached by endoscope^1^**						
Esophagus	(Reference)			(Reference)		
Stomach	0.14	(0.03 - 0.73)	0.019*	0.90	(0.33 - 2.49)	0.839
Duodenum	0.06	(0.16 - 0.22)	< .001*	0.64	(0.25 - 1.59)	0.334
**Suicide/Accident^1^**						
Suicide	4.34	(1.02 - 18.50)	0.047*	1.19	(0.92 - 1.54)	0.187
Accident	(Reference)			(Reference)		
**Systemic complications^1^**	26.44	(7.69 - 90.90)	< .001*	-		
Aspiration pneumonia^1^	1.15	(0.82 - 1.63)	0.423	0.93	(0.59 - 1.47)	0.750
Respiratory failure^1^	2.14	(1.35 - 3.39)	0.001	1.78	(0.99 - 3.22)	0.055
DIC^1^	1.38	(0.62 - 3.11)	0.434	1.18	(0.48 - 2.90)	0.724
Hepatic^1^	2.37	(1.29 - 4.36)	0.006	2.69	(1.41 - 5.13)	0.003*
GI complications^1^	2.85	(1.26 - 6.48)	0.012*	0.76	(0.54 - 1.05)	0.094
Fever^1^	7.36	(2.96 - 18.25)	< .001*	0.63	(0.39 - 1.03)	0.063
Antibiotic usage^1^	14.12	(4.13 - 48.19)	< .001*	1.87	(1.18 - 2.97)	0.008*
Psychological comorbidity^1^	1.43	(0.63 - 3.29)	0.395	-		
Systemic comorbidiy^1^	1.54	(0.60 - 3.97)	0.375	-		

^1^The age classification was adjusted to avoid selection bias due to age.

* *P *value < 0.05

DIC, disseminated intravascular coagulation

The age-related clinical confounders shown in Table 3 were analyzed further using the multivariate regression model for mortality risk. Patients with grade 3 as compared to grade 1 injury, with hepatic or systemic complications, and who used antibiotics had the greatest mortality risk related to caustic ingestion after adjusting for age and gender. Psychological comorbidities and systemic comorbidities were found to be significantly different between the two age groups but did not correlate with increased mortality risk.

**Figure 1:**
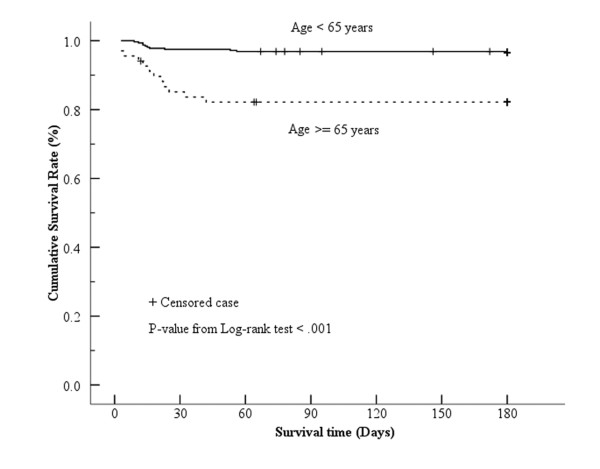
**Log-rank test Kaplan-Meier survival curves for elderly and non-elderly adults following caustic ingestion**.

Figure 1 presents the Kaplan-Meier survival curves for the elderly and non-elderly groups. The estimated two-month survival rate was 96.9% for the non-elderly group and 82.2% for the elderly group. More than 50% of subjects in this study survived during the study period. The overall estimated mean survival time was 170.9 days (95% CI = 167.1 - 174.7). The estimated mean survival time for the non-elderly group was 175.1 days (95% CI = 171.9 - 178.2) compared to 151.1 days for the elderly group (95% CI = 136.2 - 165.9). From the log-rank test, the cumulative survival rate was found to be significantly lower for the elderly as compared to the non-elderly group (*P *< 0.001).

## Conclusion

The present study is the first to demonstrate that the clinical outcome for adults who ingest caustic substances is related to age (*P *value of < 0.001 from the log-rank test; Figure 1). In addition, the incidence of systemic complications due to caustic ingestion was found in this study to be significantly higher in adults 65 years of age or greater as compared to those below 65 years of age. The increased incidence of systemic complications in the elderly group correlated with an increased mortality risk for subjects in this group (HR = 26.44; 95% CI = 7.69 to 90.90; *P *< 0.001; Table 3). Elderly subjects, those with systemic complications such as respiratory failure, aspiration pneumonia, and DIC were found to have the greatest mortality risk due to caustic ingestion.

Aspiration pneumonia is part of a spectrum of inflammatory pulmonary disorders which can increase morbidity and mortality even in anesthetized patients [11]. Caustic agents with pH values < 2 or > 12 cause different forms of mucosal injury when aspirated. In addition the esophageal mucosa, which is particularly sensitive to acidic substances, easily induces regurgitation in response to such substances [5,12], leading to aspiration pneumonia. In cases of caustic injury, aspiration pneumonia is reported to have a low complication rate (4.2%) but is associated with increased mortality (~ 60%) and occurs more commonly in older patients [13]. Aspiration pneumonia occurred more frequently in our patients as compared to those in other studies and its incidence increased with age (10.3% in the non-elderly group and 23.5% in the elderly group; *P *= 0.003).

In contrast to the increased mortality risk associated with the presence of systemic complications in the elderly group, the severity of GI tract injury appears to have had no impact on survival despite the fact that patients in this group had more severe mucosal injury (grade 3b) than those in the younger group. Upper GI tract bleeding after caustic injury is another major complication of caustic ingestion. In the present study, the bleeder rate was approximately 4.4% for all patients, regardless of age. In addition, the incidence of other GI complications such as stricture, perforation and fistula formation was also independent of age. Furthermore, based on the multivariate Cox-regression model of mortality risk considering relative age-related confounding factors, neither the type of GI complication nor the location or severity of GI tract injury appears to have an impact on survival after caustic injury in the elderly.

Certain findings of the present study are not unexpected. For example, the incidence of comorbidities such as diabetes mellitus, hypertension, major organ disease or operative history was higher for elderly subjects (73.5% for the elderly group as compared to 22.7% for the non-elderly group; *P *< 0.001). In addition, no differences were observed between the two age groups regarding the type of ingested substance, the pH of the ingested substance, or the site most severely damaged by the caustic agent.

It has been suggested [14] that endoscopy should be avoided in cases of third-degree lesions or in children without signs or symptoms after caustic ingestion. Minimally invasive management consisting of flexible endoscopy, guidewire-assisted esophageal balloon dilatation and intralesional triamcinolone injection without gastrostomy or esophageal stent placement is reported to be safe and effective for relief of dysphagia and avoids iatrogenic esophageal perforation [15]. Nonetheless EGD remains the gold-standard to assess the depth and extent of injury safely such that the appropriate therapeutic regimen can be selected [2]. According to recent studies, EGD evaluation is safe if performed within 12 h and no later than 24 h of ingestion of caustic materials. However, this procedure is not recommended after a delay of 2 to 3 days because of the wound softening that occurs at these later times [3,16,17]. In accord with the findings of other investigators [18], endoscopic classification was found useful for predicting the immediate and long-term complications of caustic ingestion and for selecting appropriate therapy for elderly subjects.

The percentage of all patients in the present study who attempted suicide was greater than 70%; similar percentages were obtained in each of the two age groups (72.9% for the non-elderly group and 70.6% for the elderly group; *P *= 0.699). This observation strongly supports the proposal that complications of the ingestion were consequences of the ingestion itself as opposed to consequences of comorbidities. It should be noted, however, that the percentage of suicide attempts in this study was higher than that reported in other studies [7,9].

Certain limitations of the present study should also be noted. Firstly, this retrospective review involved a limited number of patients in a community hospital setting. Secondly, the clinical outcomes for subjects who attempted suicide and for those who suffered grade 3b injuries were not specifically provided. Future prospective studies should focus on greater numbers of patients and include a broader range of patient settings with special emphasis on factors such as type of GI complication, location of GI injury, grade and type of GI injury, comorbidities, intent of caustic ingestion, and incidence of aspiration pneumonia.

In conclusion, the clinical outcome for adults who ingest caustic substances is age-related. The incidence of systemic complications, including respiratory failure, aspiration pneumonia, and DIC, is higher in elderly as opposed to non-elderly adults following caustic ingestion; the incidence of systemic complications in these elderly subjects correlates with their mortality risk. The severity of GI tract injury does not appear to impact the survival of elderly subjects. When the elderly suffer from caustic injury, their respiratory systems must be supported with particular care to prevent aspiration pneumonia. If infection is suspected, prophylactic administration of antibiotics is indicated and should be done as soon as possible.

## Competing interests

The authors declare that they have no competing interests.

## Authors' contributions

JMC, LYH and HTC participated in drafting of the manuscript. NJL and HTC participated in conception and design. CJP and MHT participated in analysis and interpretation of data. CSL and YYC participated in critical revision of the manuscript for important intellectual content. CCL, CTU and HTC approved the final manuscript.

## Pre-publication history

The pre-publication history for this paper can be accessed here:

http://www.biomedcentral.com/1471-230X/11/72/prepub
